# “Go ahead and screen” - advice to healthcare systems for routine lynch syndrome screening from interviews with newly diagnosed colorectal cancer patients

**DOI:** 10.1186/s13053-023-00270-4

**Published:** 2023-11-17

**Authors:** Jennifer L. Schneider, Alison J. Firemark, Sara Gille, James Davis, Pamala A. Pawloski, Su-Ying Liang, Mara M. Epstein, Jan Lowery, Christine Y. Lu, Ravi N. Sharaf, Andrea N. Burnett-Hartman, Victoria Schlieder, Zachary M. Salvati, Deborah Cragun, Alanna Kulchak Rahm, Jessica Ezzell Hunter

**Affiliations:** 1 Kaiser Permanente Center for Health Research, 3800 N Interstate Ave, 97227 Portland, OR USA; 2 HealthPartners Institute, Bloomington, MN USA; 3 Palo Alto Medical Foundation Research Institute, Palo Alto, CA USA; 4 Division of Health Systems Science, Department of Medicine, University of Massachusetts Chan Medical School, Worcester, MA USA; 5 University of Colorado Cancer Center, Aurora, CO USA; 6 Department of Population Medicine, Harvard Medical School, Harvard Pilgrim Health Care Institute, Boston, MA USA; 7 Division of Gastroenterology, Department of Medicine, Division of Epidemiology, Department of Population Health Sciences, Weill Cornell Medicine, New York, NY USA; 8 Institute for Health Research, Aurora, CO USA; 9 Geisinger Department of Genomic Health, Danville, PA USA; 10 University of South Florida, 3720 Spectrum Blvd, Suite 304, Tampa, Fl USA; 11 RTI International, Research Triangle Park, Durham, NC USA

**Keywords:** Lynch syndrome, Colon cancer, Universal tumor screening, Implementation, Patient perspective, Qualitative

## Abstract

**Background:**

Lynch syndrome (LS) is the most common cause of inherited colorectal cancer (CRC). Universal tumor screening (UTS) of newly diagnosed CRC cases is recommended to aid in diagnosis of LS and reduce cancer-related morbidity and mortality. However, not all health systems have adopted UTS processes and implementation may be inconsistent due to system and patient-level complexities.

**Methods:**

To identify barriers, facilitators, and suggestions for improvements of the UTS process from the patient perspective, we conducted in-depth, semi-structured interviews with patients recently diagnosed with CRC, but not screened for or aware of LS. Patients were recruited from eight regionally diverse US health systems. Interviews were conducted by telephone, 60-minutes, audio-recorded, and transcribed. An inductive, constant comparative analysis approach was employed.

**Results:**

We completed 75 interviews across the eight systems. Most participants were white (79%), about half (52%) were men, and the mean age was 60 years. Most self-reported either no (60%) or minimal (40%) prior awareness of LS. Overall, 96% of patients stated UTS should be a routine standard of care for CRC tumors, consistently citing four primary motivations for wanting to know their LS status and engage in the process for LS identification: “knowledge is power”; “family knowledge”; “prevention and detection”; and “treatment and surveillance.” Common concerns pertaining to the process of screening for and identifying LS included: creating anticipatory worry for patients, the potential cost and the accuracy of the genetic test, and possibly having one’s health insurance coverage impacted by the LS diagnosis. Patients suggested health systems communicate LS results in-person or by phone from a trained expert in LS; offer proactive verbal and written education about LS, the screening steps, and any follow-up surveillance recommendations; and support patients in communicating their LS screening to any of their blood relatives.

**Conclusion:**

Our qualitative findings demonstrate patients with CRC have a strong desire for healthcare systems to regularly implement and offer UTS. Patients offer key insights for health systems to guide future implementation and optimization of UTS and other LS screening programs and maximize diagnosis of individuals with LS and improve cancer-related surveillance and outcomes.

**Trial registration:**

Not available: not a clinical trial.

**Supplementary Information:**

The online version contains supplementary material available at 10.1186/s13053-023-00270-4.

## Background

Lynch syndrome (LS) is the most common cause of inherited colorectal cancer (CRC) [1]. Approximately one million individuals in the US have LS, but only about 2% know it, and hence the great majority do not receive life-saving surveillance and treatment [2, 3]. A diagnosis of LS can impact clinical and cancer surveillance recommendations for patients and their relatives [4, 5]. Universal tumor screening (UTS) of all newly diagnosed CRC cases to identify patients at high risk for LS, with follow-up germline genetic testing to confirm the diagnosis, is recommended for all individuals with CRC [1, 6–8]. UTS has been adopted by many, but not all, healthcare systems across the US [4, 9], and even among those with a UTS program, implementation of screening guidelines are inconsistent [10].

Despite the promise of UTS to increase diagnosis of LS and reduce cancer-related morbidity and mortality, the screening process poses potential barriers. At the healthcare system level, UTS programs may be costly, involve coordination across multiple departments, and may compete with other priorities [1, 11–14]. Several different screening options exist; [15] most involve a two-step process of first testing tumors for mismatch repair deficiency, the pathologic hallmark of LS, and second, referring patients with a positive tumor screening result for germline genetic testing to confirm LS [1]. This multi-step process requires all individuals with a positive screen to be contacted and referred for genetic testing. Further, patients need information to help them understand the implications of a diagnosis of LS for themselves and their families and to help guide decisions about receiving germline genetic testing.

Studies show that most patients have not heard of LS or UTS programs, and rely on their providers or healthcare system to facilitate screening and testing [6, 16, 17]. In previous studies, both patients and providers have supported consistently screening for LS, but both have also questioned how to handle patient communication, consent, privacy, costs, and sharing screening results with family members [6, 10, 18–22]. Given inconsistent implementation of UTS programs and ongoing questions regarding LS screening, healthcare systems could benefit from in-depth patient perspectives on the value of receiving this genetic risk information and on implementing screening programs to address the concerns of patients newly diagnosed with CRC.

We explored the perspectives of patients recently diagnosed with CRC across multiple healthcare systems with and without UTS programs to uncover reactions to the idea of screening for LS and barriers and facilitators to UTS programs. In-depth, semi-structured qualitative interviews were conducted as a secondary aim as part of the main IMPULSS (Implementing Universal Lynch Syndrome Screening) study. The overall IMPULSS study seeks to identify facilitators and barriers to optimal LS screening implementation in healthcare settings, and has been described elsewhere [23]. Our patient interviews provide additional important patient perspectives that can inform future implementation and optimization of UTS programs as well as other LS screening approaches such as those that offer germline testing without the initial tumor screen.

## Methods

### Study setting/background

Patients recently diagnosed with CRC were identified from the eight participating healthcare systems in the main IMPULSS study, representing diverse geographic regions across the U.S. The healthcare system sites varied in size, location, and degree to which they had standard practices for screening patients for Lynch syndrome. Patients newly diagnosed with CRC were chosen as the target population for the interviews given this is the timeframe and context in which tumor screening for Lynch syndrome would typically occur within a health system. At the time of our interviews (September 2018 to early March 2020), five of the systems had existing UTS programs and three did not. Two sites were integrated healthcare systems where all patients are members, while the other five sites were “open” systems where members receive care in or outside the system, and patients who are not members of the health plan can receive care. The eighth site was a faith based, not-for-profit healthcare system providing care in 18 states. Additional information about the main IMPULSS study and participating sites has been previously reported [23].

### Recruitment

Interviews were conducted centrally at one healthcare system by a consistently staffed expert qualitative team (JLS, AJF, JD) after local-level recruitment at each site. To recruit patients, each site identified potentially eligible patients 18 + years of age (no upper limit) with a CRC diagnosis with adenocarcinoma histology within the previous 60 to 150 days and no prior LS diagnosis or screening evidence. All stages of CRC were included. Patients were all English-speaking. Patients with a prior CRC or LS diagnosis were excluded, as were individuals residing in assisted living or those with a dementia diagnosis. Chart abstraction confirmed exclusion criteria and the patient’s adenocarcinoma histology.

Eligible patients received an invitation by mail including a detailed description of the study. Study participation was voluntary, and patients could opt in or out by contacting local study staff. Outreach protocols were tailored to meet the requirements of each healthcare system. Two systems with opt-in protocols asked interested patients to first contact local site research staff to volunteer for interview participation. Patient contact information was then shared with the qualitative team. At the remaining five systems with an opt-out protocol, patient contact information for those who did not opt-out was shared with the qualitative team, who then contacted participants by telephone to invite them to schedule an interview. All participant information was shared via an approved, encrypted secure data transfer process. Our goal was to interview 8–10 patients per site to obtain patient perspective from each health system site participating in the main IMPULSS study, and to generate an overall interview sample size sufficient for thematic analysis. Eligible patients were approached at each site until the goal was reached or no additional eligible patients were available. The recruitment process lasted approximately 19 months, as each site was recruited and completed before moving onto the next site, with opt-in sites taking the longest to complete.

### Data collection

The qualitative team developed an in-depth, semi-structured interview guide for use with participants diagnosed with CRC and without an existing LS diagnosis. We solicited feedback on the interview guide from investigators at each site, from an external clinical expert in screening for LS, and from a patient advocate for LS screening, and revised the guide in response to their input. Given we wanted to obtain novice reaction to the UTS program process of screening, participants were intended to be naïve to LS. Hence, the guide hypothetically explored reactions and motivations for learning about one’s LS status, including the two-step process of first screening the tumor for markers of LS, followed by referral for confirmatory genetic testing. Interview areas included: general awareness of LS; reasons one may want to learn if they have LS; motivations for and concerns about LS screening steps (tumor screening followed by confirmatory genetic testing); preferences for communicating LS screening and diagnosis results; and overall suggestions for health systems regarding UTS programs.

At the end of the interview, participants were asked to self-report on demographic characteristics. Participants were also asked about personal and family histories of cancer and whether they had family members who received care in the same healthcare system. Finally, to further describe the study population, participants were asked about their preference for receiving an explanation using either words or numbers to describe the ‘risk of something happening’ [24] and to assess health literacy [25] (Supplementary material, interview guide).

Interviews were conducted and audio-recorded via telephone by the qualitative team (JLS, AJF, JD), with over 20 years of experience and training in qualitative data collection and analysis. To ensure consistency in data collection, the qualitative team met regularly (twice monthly and ad-hoc as needed) during the interviewing process to discuss application of the interview questions and make any needed adjustments to how questions were asked. Interviews averaged 60 min, and participants received a $25 gift card for their participation. Interviews were conducted between September 2018 and early March 2020 (prior to the national stay at home orders due to the COVID-19 pandemic), and all interview procedures and materials were IRB-approved.

### Data analysis

All recorded interviews were professionally transcribed verbatim for analysis. Using an inductive approach, the qualitative team (JLS, AJF, JD) reviewed a random sub-sample interview transcripts and developed a preliminary codebook. Input was provided by the larger study team and the codebook was revised accordingly. During the coding and analysis process, the qualitative team met regularly (twice monthly and ad-hoc as needed) to discuss consistent application of codes, codebook refinement and re-application of codes as needed, and summarization. Coding and content analysis was aided by NVivo 12.0, a qualitative analytic software tool [26]. Next, reports of coded text were generated and grouped into common topical areas (e.g., reasons to obtain tumor screening for LS) by the qualitative team for further summarization (Supplementary material, topical areas and codes), again meeting regularly to discuss and document interpretation. A constant comparative approach was applied to synthesize findings into comprehensive themes [27–31]. Theme reports were developed and presented to the larger study team for input. The qualitative team reviewed original transcripts when necessary and integrated feedback into the next iteration of theme reports. Preliminary findings were also shared with our patient advocate for reaction and discussion. This ongoing member-checking process was an integral part of the analytic process, helping the qualitative team more accurately interpret and triangulate the emerging themes [27, 28, 32]. Our iterative process led to the development of specific summaries of interview data for each healthcare system, categorized by themes within key topical areas. While healthcare systems were purposively selected for the main IMPULSS study as having a UTS program or not, participant interviews about UTS programs and the screening process were not thematically different across sites; therefore, data were analyzed across all interviewed participants. Hence, site-specific summaries were reviewed with the larger study team and then further collapsed into overarching key themes across all sites representing all participants, forming the data for this manuscript. As part of our analysis, transparency, and data display process, we include the number and percent of participants that acknowledged themes (e.g. %/number of participants with accuracy concerns), [33–35] as well as provide rich-description through participant quotes [27–29]. Additionally, the Consolidated Criteria for Reporting Qualitative Research (COREQ) was employed to guide rigor and presentation of our findings [36].

## Results

### Participant description

We completed 75 interviews across the eight healthcare systems, with ten at most sites (range 6–11). Interviewers contacted 282 individuals; 138 were unreachable after multiple attempts, 64 actively declined participation, and five scheduled an interview and later declined. Table 1 describes the characteristics of interview participants. More than half (52%; 39/75) were men, and the mean age was 60 years. Most had private insurance (93%; 70/75), and one-third (36%; 27/75) reported having a child seen by the same healthcare system. The majority were white (79%; 59/75) and married or living with a partner (72%; 54/75). While only 17% (13/75) self-reported a personal cancer diagnosis prior to their current CRC, more than half (56%; 42/75) self-reported a family history of any cancer. Most participants indicated adequate health literacy, with 88% (66/75) rating their confidence in filling out forms as quite a bit or extremely confident. Further, 63% (47/75) preferred numbers over words when receiving an explanation of the chance of something occurring. Finally, 60% (45/75) self-reported no knowledge or awareness of LS prior to our interviews. The 40% (30/75) who were aware of LS typically had minimal knowledge.

**Table 1:** Participant descriptive characteristics (*N* = 75)

Characteristic	Value	(%)
Age	Mean: 60 years	
	Range: 26-86 years	
Gender	Female	48%
	Male	52%
Insurance	Private	93%
	Medicaid only	3%
	Medicare only	3%
	None	1%
Patient-reported race/ethnicity	White	79%
	Asian	4%
	Black	4%
	Hawaiian	1%
	Multiple^a^	12%
Household income	<$15,000	4%
	$15-30,000	15%
	$30-50,000	20%
	$50-75,000	7%
	$75-100,000	11%
	>$100,000	31%
	Prefer not to answer	4%
	Do not know	1%
Highest level of education	Some high school	3%
	High school	16%
	Trade school	4%
	Some college	20%
	College graduate	28%
	Postgraduate	29%
Married or living with partner	Yes	72%
	No	28%
Family members receiving care from same health system	None	56%
	Child(ren)	34%
	Sibling(s)	5%
	Child(ren) and cousin	1%
	Child(ren) and grandchild(ren)	1%
	Nieces and nephews	1%
Prior personal cancer diagnosis	Yes	17%
	No	83%
Prior family history of cancer (blood relatives)	Yes	56%
	No	44%
Confidence in filling out forms (health literacy)	Extremely	56%
	Quite a bit	32%
	Somewhat	8%
	A little bit	3%
	Not at all	1%
Prefer words or numbers when assessing “chance” of something (health risk)	Numbers	63%
	Words	21%
	Either	15%
	No response	1%
Prior knowledge of Lynch Syndrome	None	60%
	Some knowledge/awareness	40%

^a^Includes 4 White and Middle Eastern; 1 White, Hawaiian, and Asian; 1 White, Laplander, and Asian; 1 White, American Indian and Hispanic; 1 White and Celt; and 1 American Indian and Hispanic

*“I don’t know anything about genes that could raise the risk of cancer…Today is the first day I’ve heard about Lynch Syndrome, so I don’t think that they’ve [healthcare system] ever done anything on it.” –* Site7 patient.

*“I did know about Lynch Syndrome because a friend of mine had just had colon cancer and she said she had Lynch Syndrome.” –* Site8 patient.

**Fig. 1:**
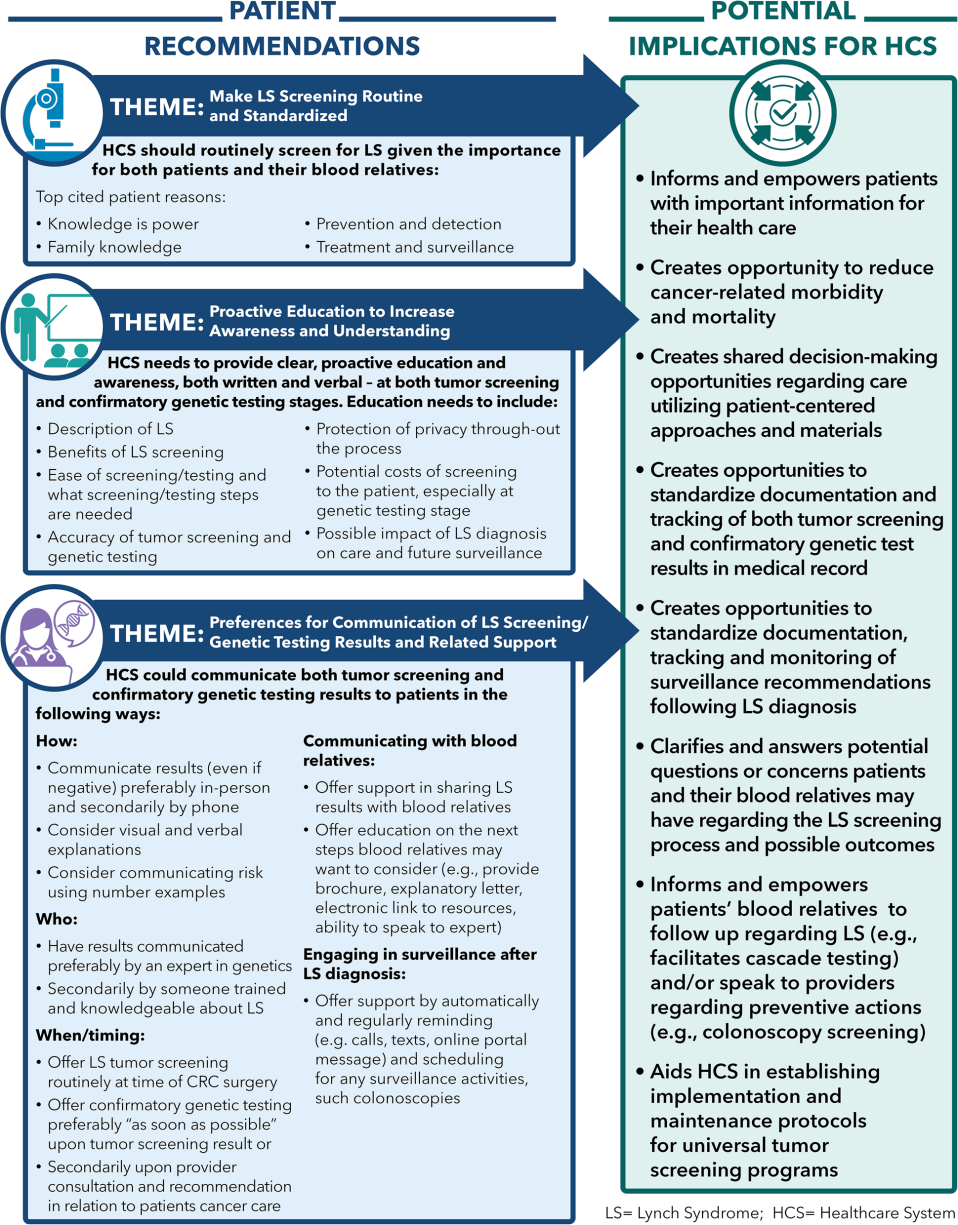
Patient suggestions and potential implications for healthcare systems regarding screening for lynch syndrome

We divide our results into the following areas, highlighting motivations and concerns within each: (1) initial reactions to the idea of learning about one’s LS status; and (2) reactions to the steps in UTS for LS, including tumor screening and follow up confirmatory genetic testing. Table 2 summarizes patient motivations and Table 3 summarizes patient concerns. Both tables display the level of patient endorsement (%/n) of a motivation or concern at the three stages of initial reaction, tumor screening, and confirmatory genetic testing. Finally, we identify patient communication preferences and overall advice for healthcare systems to consider when implementing a UTS program (Fig. 1).

**Table 2:** Summary of motivations at different stages for learning lynch syndrome status (N = 75; Bolded % indicates most common endorsements)

THEMES: Reasons and motivations to learn about Lynch Syndrome (LS) and obtain screening status	Topic: Initial Reactions - General motivations to know LS status % (n)	Topic: Step 1 - Motivations to have Tumor Screening % (n)	Topic: Step 2 - Motivations to have Genetic Test % (n)
**“family knowledge”** – important to inform blood relatives	**68% (51)**	**41% (31)**	**61% (46)**
**“knowledge is power”** – necessary and important health knowledge for self	**67% (50)**	**63% (47)**	**96% (72)**
**“prevention and detection”** – helpful for identifying and preventing possible future cancers or reoccurrences	**64% (48)**	**56% (42)**	**56% (42)**
**“treatment and surveillance”** – helpful for possibly informing treatment decisions and monitoring actions	**55% (41)**	**49% (37)**	**53% (40)**
**“helpful for future research and patients”** – help with science and other similar patients	-	11% (8)	5% (4)
**“explain personal and/or family cancer history”** – helps fill in possible knowledge gaps or curiosity as to “why”	-	5% (4)	-
**“provider recommendation”** – important for conveying importance of learning LS status and fostering follow-through	-	-	16% (12)
**“seek additional research and information”** – conduct own information gathering to further understanding and actions	-	-	9% (7)
**“attending additional appointment”** – not a concern or barrier for obtaining follow-up genetic testing	*	*	**87% (65)**
**“privacy and documentation”** – not a concern or barrier for LS status to be documented in medical record	*	*	**88% (66)**

*Notations:* - indicates theme did not naturally come up during this point in the interview; * indicates topic area not explored at this point in the interview

**Table 3:** Summary of concerns at different stages for learning lynch syndrome status (*N* = 75; Bolded % indicates most common endorsements)

THEME: Concerns or barriers that may prevent learning Lynch Syndrome (LS) status or obtaining LS diagnosis	Topic: Initial Reactions - General motivations to know LS status % (n)	Topic: Step 1 - Motivations to have Tumor Screening % (n)	Topic: Step 2 - Motivations to have Genetic Test % (n)
**“no barriers”** – generally no concerns or reasons to forgo learning LS status or follow through with screening steps	**64% (48)**	**61% (46)**	**41% (31)**
**“worry and anxiety”** – concern that knowing or learning information may generate needless stress for patient or prolong cancer journey	**35% (26)**	9% (7)	4% (3)
**“unnecessary information”** – given older age, lack of family history, cancer trajectory, personal beliefs, or mobility issues	8% (6)	3% (2)	9% (7)
**“does not gain anything medically actionable”** – if knowing LS status didn’t change anything about treatment options or surveillance or future actions	5% (4)	-	-
**“cost challenges”** – concern if high cost, or high co-pay, or not covered well or at all by insurance, lack of cost information generates hesitation	4% (3)	9% (7)	**79% (59)**
**“impacts health insurance coverage”** – concern LS diagnosis could impede ability to obtain future health insurance coverage	4% (3)	**19% (14)**	12%; (9)
**“privacy concerns”** – concern LS diagnosis could impact future care or services in future/ sensitive personal information	4% (3)	3% (2)	-
**“patient informed choice”** – concern some patients may desire shared-decision making and consent for pursuing steps in screening	-	12% (9)	4% (3)
**“accuracy of testing”** – concern over level of accuracy, certainty and trustworthiness of a potential LS diagnosis	-	-	**23% (17**)
**“attend an additional appointment”** – inconvenience of a second appointment may be a barrier for obtaining follow-up genetic testing	*	*	13% (10)
**“no provider recommendation”** – lack of provider recommendation regarding LS screening may limit interest or sense of importance	3% (2)	1% (1)	9% (7)

*Notations:* - indicates theme did not naturally come up during this point in the interview; * indicates topic area not explored at this point in the interview

### Initial reactions

After providing a brief lay-person description of LS, we assessed initial reasons why participants might want to learn about their LS status. Four consistent and primary motivations arose: (1) to inform blood relatives for their own health (“family knowledge” − 68%; 51/75); (2) to know and want as much health information as possible (“knowledge is power” − 67%; 50/75); (3) to prevent and detect future cancers (“prevention and detection” − 64%; 48/75); and (4) to potentially inform treatment decisions and cancer surveillance actions (“treatment and surveillance” − 55%; 41/75). Participants representing all eight healthcare systems identified these reasons with most noting multiple motivations.

*“I think it’s important to know. I would want to know for my kids’ sake so they could get tested and be prepared to deal with things that may come their way.” –* Site5 patient.

*“For me it’s really related to making sure I am being monitored in the right way. And it seems like a diagnosis of Lynch Syndrome very much changes the way and the frequency with which you’re monitored for a variety of different cancers - I would find it very important to know that.” –* Site4 patient.

When reflecting on reasons to generally not learn one’s LS status, most participants (64%; 48/75) stated there were “no reasons” or concerns to forego learning or knowing this information.

*“There’s no reason why I wouldn’t want to know…because if you know it you’re more likely to follow-up on all the tests you need to have. So, I think it’s good to know.”–* Site7 patient.

Some participants identified minor concerns about potentially learning their LS status. The possibility of generating needless “worry or anxiety” was a concern for about one-third of participants across all eight systems (35%; 26/75). Less frequently mentioned concerns at this time point included: perceived as “unnecessary information” given age or cancer diagnosis (8%; 6/75); if knowing LS status “did not gain anything medically actionable” (5%; 4/75); if it created “cost challenges” (4%; 3/75); if it negatively “impacted health insurance coverage” (4%; 3/75), or generated “privacy concerns” (4%; 3/75).

*“This could be another thing to add to the fire of anxiety. If there’s no treatment for it, then what good is it.” –* Site5 patient.

*“I don’t feel that it’s even urgent for me. After all I’m eighty-two years old.” –* Site1 patient.

*“You know, [if] all of a sudden, I’m not able to get certain kinds of insurance, I get labeled a certain thing from a medical perspective…that is concerning.” –* Site3 patient.

### Reactions to screening steps

Participants next reflected on possible motivations and barriers to common process steps in UTS programs: (1) tumor screening, typically performed automatically by the healthcare system and does not require patient-level decision-making; and (2) confirmatory germline genetic testing, which requires the patient to decide whether to follow up to confirm the diagnosis of LS. Although both steps impact the patient, how each step is completed is different. Thus, it was important to capture perspectives on each step separately to determine whether patients understand and value the information gained at each step and the role they and their healthcare system have in this process.

*Step 1: Tumor screening*. Participants across all systems identified the same four primary motivations for wanting their CRC tumor biopsied and screened for LS as they did for generally wanting to learn their LS status, including: “knowledge is power” (63%; 47/75); “prevention and detection” (56%; 42/75); “treatment and surveillance” (49%; 37/75); and “family knowledge” (41%; 31/75). Participants identified additional reasons, including believing it to be “helpful to future research and patients” (11%; 8/75); and that it may help “explain personal or family history of cancer” (5%; 4/75).

*“Being able to have that test and knowing whether or not I have Lynch Syndrome, it’s valuable because if I did know, I could prepare myself more.” –* Site2 patient.

*“Just to protect myself for the rest of my life. And also, to just keep my boys on top of things and as they get older, make sure that they’re doing the right thing.” –* Site8 patient.

Similar to generally wanting to know their LS status, 61% of participants (46/75) stated there would be “no reasons” or meaningful barriers to wanting tumor screening performed. However, a few concerns were identified. The possibility of negatively “impacting health insurance coverage” (19%; 14/75) and “cost challenges” (9%; 7/75) both came up slightly more often when reflecting on the tumor screening step. A new concern emerged for a small set of participants (12%; 9/75) regarding whether “patient informed choice” would be offered and these individuals suggesting shared decision-making be considered at this tumor screening step. Thoughts about generating “worry and anxiety” (9%; 7/75), “privacy concerns” (3%; 2/75), and “unnecessary information” due to older age (3%; 2/75) came up less as concerns when considering the tumor screening step.

*“It’s very important to know -I don’t have any problem with the tumor being tested.” –* Site4 patient.

*“The only thing that would hurt me would be like for insurance purpose - if it was held against you at a later time…because if it’s genetic there’s nothing you can do about it.”–* Site6 patient.

*“You know, having some choice [shared-decision-making] is always good.”* – Site3 patient.

*Step 2: Confirmatory genetic test.* Participants next reflected on following through with obtaining a genetic test. Motivations to have the genetic test mirrored the same four reasons for tumor screening and general knowledge of LS status across participants from all eight systems, including: “knowledge is power” (96%; 72/75); “family knowledge” (61%; 46/75); “prevention and detection” (56%; 42/75); and “treatment and surveillance” (53%; 40/75).

*“I’m willing to do that [second step], whatever I need to do…I want to know. And I have children and grandchildren and siblings that I would like them all to be aware.” –* Site7 patient.

Two new motivations emerged from participants reflecting on this stage of confirmatory genetic testing, including having a “provider recommendation” (16%; 12/75) and a desire to seek out additional “research and information” on the topic (9%; 7/75). A few noted they would be motivated by the possibility that it could be “helpful to future research and patients” (5%; 4/75).

“*I’d likely have it done… And I’d ask my doctors, ‘what’s the best thing we could do?’ - whatever [surgeon] tells me I will listen because it’s the best not only for me, it’s best for my kids.” –* Site2 patient.

Regarding the possibility of having to make and “attend an additional appointment” to obtain the confirmatory genetic test, 87% (65/75) of participants representing all sites did not perceive that as a barrier. Additionally, most participants (88%; 66/75) had no concerns about “privacy or documentation” of the genetic test finding in their medical record. 41% of participants (31/75) stated there would be “no reason” to forgo the confirmatory genetic test.

“*It sounds like a great idea. And it’s a simple one [for attending additional appointment]. I mean it is not invasive. It’s just taking blood… I need to know one way or the other*.” – Site4 patient.

“*I think it should be in the medical records – everything is on the computer; everything is right there. It’s an important thing…to have everything on file in one location.” –* Site1 patient.

When concerns were noted by participants, possible “cost challenges” emerged as the most common for step 2 of confirmatory genetic testing. While 21% (16/75) had no concerns regarding potential costs deterring follow through, 79% (59/75) expressed cost questions or concerns. Of these 59 participants, 68% (40/59) stated knowing the cost upfront would be important for planning, but would likely not deter follow through with the genetic test, given they view it as necessary to their health and describe themselves as having “good insurance.” Due to their recent cancer care and treatment experiences, these participants described the importance of proactively understanding medical costs, given that recommended tests and procedures can be expensive and are not always covered well by health insurance. These 40 participants believed knowing upfront whether the cost would be “prohibitively expensive” (e.g., $5000), would be important to both financial planning and the timing of when to complete the confirmatory test in terms of meeting insurance deductibles and authorizations.

*“Cost is always out there. But some things are important…I don’t really want to pay for a $2,000 blood draw, but if it’s reasonable [and] this is prevention…Our health plan is rather good, but we have to get authorizations, I would think that they would approve it.” –* Site2 patient.

*“If it’s going to be a $10,000 test out-of-pocket, then that becomes a practical question. The CAT Scan [for cancer care] was about $1,700, but that was part of my deductible. And I actually didn’t ask before what the cost was. But in that context, I want to be aware of both the deductible and max out of pocket in advance – [it’s] a question one needs to ask.”* – Site3 patient.

For the remaining 19 of 59 (32%) participants, the potential cost of the genetic test could be a reason to decline it due to their current financial or health insurance status. Most of these participants described how their recent cancer treatment had drained their finances, with some still paying for their cancer care. Additionally, some of the 19 described being on a fixed income or experiencing a drop in their income level (i.e., job loss, retirement) that would make paying out of pocket burdensome. Others described their current insurance as a possible barrier for covering the cost of genetic testing, such as being on a high-deductible plan or concern that Medicare may not cover the genetic test. For all these reasons, these 19 participants stated any genetic testing costing over a few hundred dollars would be a financial challenge that could deter follow through.

*“It would have a high impact on my decision, depending on the cost. It’s hard to put a number on that. When you’re older and retired and your income is fixed, cost is important. For selfish reasons I would want it [genetic test] to be as low as possible. And being under Medicare, I would want them to cover it.” –* Site5 patient.

*“I do worry [about costs] because my spouse is working two jobs right now where we’re trying to pay hospital bills and stuff. I’ve got payments for surgery last year. I would want to know [cost of genetic test for LS]. If it’s real high I wouldn’t have it done because I don’t want to put no more burden …That is a concern because some insurances don’t pay for some stuff.” –* Site6 patient.

The second most common concern described by 23% (17/75) of participants centered on the trustworthiness and “accuracy of testing”. For example, participants felt if the genetic test was less than 50% accurate in confirming LS, then that might impact their willingness to follow through.

*‘If there’s only 20% chance that it’s accurate, then that might weigh on my decision of, ‘is this worth the time and effort, etcetera?’” –* Site1 patient.

Having to “attend an additional appointment” was a concern for 13% (10/75) of participants, particularly as it pertained to possible transportation challenges and the “timing” of the genetic test relative to other ongoing cancer treatment. Additionally, some participants (12%; 9/75) were concerned about the test result “impacting health insurance coverage,” fearing documentation of the result could lead to insurance discrimination.

*“If I couldn’t get down there [for genetic test] …being too old to drive or if I couldn’t see well enough…”* – Site8 patient

*“I’d hate to see someone not get insurance because of a pre-existing condition because they were tested for it.”* – Site5 patient.

Finally, a few participants had minor concerns regarding the genetic test, including: “unnecessary information” due to older age or cancer stage (9%; 7/75); lack of “provider recommendation” about it (9%; 7/75); result may create additional “worry and anxiety” (4%; 3/75); and lack of “patient-informed choice” (4%; 3/75).

### Communication preferences and advice to health systems (Fig. 1)

Participants shared how they would prefer to learn about their potential tumor screening and follow-up genetic testing results. Over half, 51% (38/75), felt an in-person conversation would be best when sharing either tumor screening or confirmatory genetic testing results. Communicating results via phone was the next best option (25%; 19/75). Some reported that a secure email or letter communication would be acceptable (12%; 9/75) or had no strong preference (12%; 9/75).

*“I would prefer that I get news [TS or GT results] in-person. That way I can ask all the questions I need to ask right then and there.”* – Site3 patient.

Regarding whom should communicate the tumor screening and confirmatory genetic test results, 56% (42/75) suggested it should be an expert in genetics who can clearly answer patient questions and provide follow-up recommendations. Almost a quarter had “no preference,” as long as the provider is knowledgeable about LS (24%; 18/75). A few thought it should be “whoever ordered the screening” (13%; 10/75) or their current main provider, whether a primary care provider (PCP) or oncologist (6%; 5/75).

*“For me the important thing would be someone who would be capable of answering all my questions …What’s the odds of me getting cancer again, or what follow-up treatment should I be having? How often should I be going in for tests? What tests? All those kind of questions. So as long as the person had the answers, sufficiently knowledgeable, then that would be a good person.”* – Site8 patient.

Regarding when to communicate tumor screening and/or confirmatory genetic testing results for LS, almost half of the participants (43%; 32/75) believed this should be as soon as possible given the importance of the information for informing future actions, while another 41% (31/75) had no strong preference for timing, deferring to providers as to when results should be made available to patients. A few participants (8%; 6/75) suggested the result communication for either tumor screening or confirmatory genetic testing should occur after some time has passed post-cancer surgery or treatment, or had no opinion (8%; 6/75).

*“I think they should test the tumor right then and there, while they were doing this pathology report. I think the sooner you understand what’s going on and what’s coming along in your future, the better.” –* Site1 patient.

Participants offered additional suggestions. The majority, 96% (72/75), recommended healthcare systems should routinely screen for LS given the importance for both patients and their blood relatives. In doing this, participants suggested healthcare systems need to provide clear, proactive education – both written and verbal – on the reasons for LS screening and the steps in the process, from tumor screening to confirmatory genetic testing. For example, participants suggested highlighting the following: benefits of screening; ease of screening; accuracy of results; how privacy is protected; how results potentially impact care and surveillance; and what the potential cost may be. Some participants (21%; 16/75) recommend a shared decision-making approach at the point of confirmatory genetic testing, allowing patients agency in determining whether they want to undergo genetic testing, which is consistent with the current practice of patients meeting with a genetic counselor prior to genetic testing.

*“Make it clear to the patient that they were screened and what the screening determined - what the screening was, how it works, how the results and analysis were determined - to fill in the gaps between the whole screening piece of it.” –* Site7 patient.

*“I personally think it should be automatic…I wouldn’t want to just be given the information and then dropped there. I’d want to know what I should be doing now. For instance, if [results] said ‘I don’t have it’, would that cut down on the other kinds of procedures like colonoscopies and the frequency thereof?” –*Site2 patient.

Most participants (89%; 67/75) would want support from their healthcare system in sharing their LS diagnosis result with their blood relatives, including access to trustworthy educational material regarding what LS is, how to screen for it, and what the next steps are for any family members that could be easily shared (e.g., brochure or online link). Similarly, 63% (47/75) believe healthcare systems should regularly and automatically remind and schedule patients for any ongoing surveillance following an LS diagnosis, such as scheduling yearly colonoscopies. Others advised improving provider education about LS to increase their awareness of it for their patients (11%; 8/75).

*“Either pamphlets or a letter, what it entails, what it involves so that family members have an understanding…in some of these things you need to read it a couple of times to fully grasp it.” –* Site4 patient.

*“A reminder would be helpful, a memory reminder two to three months before colonoscopy. It [colonoscopy] takes some planning.” –* Site8 patient.

## Discussion

Our qualitative findings suggest patients with CRC have a strong desire for healthcare systems to implement and offer UTS, with 96% stating it should be a routine standard of care for CRC tumors. Patients consistently cited four primary motivations for wanting to know their LS status and engage in the two-step process for LS identification (tumor screening followed by confirmatory genetic testing): “knowledge is power”; “family knowledge”; “prevention and detection”; and “treatment and surveillance.” These patient perspectives offer key insights for healthcare systems to consider when developing a UTS program for LS.

Having to attend an additional appointment for follow-up testing after tumor screening and having the LS result documented in one’s medical record were not perceived by most of our participants as barriers or concerns. However, our participants described concerns that healthcare systems may need to consider, including: creating possible worry for patients, the cost and the accuracy of the genetic test, and having one’s health insurance coverage impacted by the LS diagnosis. These results are consistent with prior studies that identified cost and coverage concerns as possible perceived barriers to pursuing screening [4, 6, 19]. Healthcare systems can mitigate these concerns. For example, they can use knowledgeable providers, such as genetic counselors or others trained in screening for LS, to clearly explain the personal and family health benefits of knowing whether one has LS, the diagnostic accuracy of confirmatory genetic testing, and address cost-related concerns and questions given that the costs of genetic testing are often covered by insurance. Education on these topics could alleviate anxiety and help patients make informed decisions to pursue confirmatory genetic testing. Indeed, support from a patient navigator, informed provider, and/or genetic counselor has been identified as a facilitator to pursue confirmatory genetic testing in prior studies [16, 17, 37].

Our participants strongly identify the importance of knowing upfront the cost of the genetic test so they can plan accordingly and not be surprised by the cost or have the cost deter follow through with the confirmatory genetic test. Healthcare systems can proactively explain to patients their potential coverage and benefits for the genetic test and provide referrals to programs or resources that could assist with costs. Additionally, given concerns about loss of health insurance, healthcare systems can proactively explain how privacy and insurance coverage are protected, such as by providing information about the Genetic Information Nondiscrimination Act (GINA) of 2008, which protects individuals from having their genetic information impact their ability to procure or maintain health insurance or employment.

Along with addressing concerns such as cost and accuracy, our participants offered specific suggestions for healthcare systems to consider when implementing or improving their UTS program (Fig. 1). At both tumor screening and confirmatory genetic testing stages, participants want clear, concise verbal explanations and written materials about LS, how and why it is screened for, the meaning of the results, and implications for future care and surveillance for themselves and their blood relatives. About half (51%) preferred their LS result at both stages in the process to be communicated in-person given the complex nature of the finding. However, given increased patient acceptance of telehealth options (phone or video), both generally and in genetic services to deliver care during the COVID-19 pandemic, [38–40] patients may now be more open to telehealth options when receiving LS results. Notably, what was more important to our participants was receiving the LS result (whether tumor screening or confirmatory genetic testing result) from a well-trained, knowledgeable expert in LS who can clearly educate on the topic and answer questions rather than from someone with more generalized knowledge (e.g., PCP). This is important to note for healthcare systems who are concerned about having a genetics provider disclose results even when they have not previously been involved in the care of these patients. Additionally, our participants strongly desired assistance from their healthcare system in communicating LS results and related implications to their blood relatives, and well over half (63%) would want the assistance in reminding them about and facilitating any needed surveillance activities (e.g., yearly colonoscopy) based on a LS diagnosis. Our findings are consistent with and expand upon other studies that have found patient and provider education on LS as a possible influence on the decision to pursue genetic testing, [4, 6, 17, 22] and that patients believe LS screening is important and are generally willing to share confirmatory test results with blood relatives [6, 9, 18, 22].

Our interview data has some limitations. Given that recruited patients were intentionally naive to LS, they were responding hypothetically to our exploration of motivations and barriers rather than reporting an actual experience. Additionally, the complexity of both interviewer explaining and patient understanding the two-step process of a UTS program and differences between tumor screening and confirmatory genetic testing may have hampered some participants’ responses. Patients who agreed to be interviewed may have had a more favorable view of screening for Lynch syndrome or may have been inclined to “please” the interviewer rather than share their true opinion (e.g. social desirability bias). However, the overall number of interviewees representing a range of healthcare systems and geographic locations, use of experienced interviewers and a semi-structured guide with instructions for interviewers, and an iterative, robust analytical process, helps to mitigate these limitations. Despite the range of healthcare systems and geographic locations, our study population was 79% white and 93% had private health care, which may limit the generalizability of our findings. While the overall number of patients interviewed allowed us to observe thematic saturation and is sufficient for qualitative content analysis, [29, 41] our findings may not represent the full range of patient opinions about health systems screening for Lynch syndrome or implementing a UTS program given the lack of socio-economic, racial, and cultural diversity of our sample.

Future studies could expand on the findings of this study. The barriers and facilitators identified could be used to guide the development of patient educational materials for different timepoints in the LS screening process, and/or to assist patients in making informed decisions on whether to seek confirmatory genetic testing. Perspectives in underrepresented or under-insured populations could be captured to ensure that UTS programs are equitably implemented and do not exacerbate existing inequities. Quantitative or survey-based approaches could be applied to characterize the frequencies of potential barriers identified in this study (e.g., out of pocket costs) in patients across a wide range of backgrounds. Patient perspectives and experiences could be assessed in patients who underwent tumor screening and either did or did not follow-up with confirmatory genetic testing to capture pragmatic barriers and facilitators. Finally, future research in this area may want to employ patient-based frameworks such as the Theoretical Framework of Acceptability (TFA) [42] to further identify both theoretical and practical implications for implementing UTS programs.

## Conclusions

It is important to note the changing landscape in healthcare related to the diagnosis of LS in LS-associated cancers. Recently, the National Comprehensive Cancer Network (NCCN) updated their guidelines to recommend going straight to germline testing for patients with CRC under the age of 50 and considering this approach for older patients with CRC, potentially limiting the frequency of tumor screening for the identification of LS. [43] However, the patient perspectives captured by this study remain applicable in the context of shifting approaches for diagnosis of LS and identify how some patients believed it was important that they be allowed to make informed decisions on whether to pursue genetic testing, consistent with the current process of receiving genetic testing.

Our findings provide key insights into patient perspectives about the importance of LS screening and related educational needs. These insights can guide health systems’ future implementation of and/or improvement to UTS programs to maximize the diagnosis of individuals with LS and improve cancer-related morbidity and mortality.

### Supplementary Information

**Additional file 1.** Interview guide (Supplementary material for publication).

**Additional file 2.** Topical areas and related codes (Supplementary material for publication).

## Data Availability

The qualitative datasets (interview transcripts and coded text from transcripts) generated and analyzed during the current study are not publicly available because they were generated from interviews conducted by the research team, with the expectation that participant identity would be kept confidential. The interview guide used for data collection is shared as supplementary material. The qualitative codebook (definitions of codes used to aid in qualitative analysis) are available upon request from the corresponding author on reasonable request.

## Abbreviations

LS: Lynch syndrome
CRC: Colorectal cancer
UTS: Universal tumor screening
IMPULSS: Implementing universal Lynch syndrome screening
