# Supplementary material for: “Go ahead and screen” - advice to healthcare systems for routine lynch syndrome screening from interviews with newly diagnosed colorectal cancer patients
Source: Hered Cancer Clin Pract. 2023 Nov 17;21:24. doi: 10.1186/s13053-023-00270-4 (PMC10657118; doi:10.1186/s13053-023-00270-4)
Supplement: Supplementary file 1 — Additional file 1. Interview guide (Supplementary material for publication). [file 13053_2023_270_MOESM1_ESM.docx]

**Patient interview guide**

***Hello, my name is __________ and I am from the Kaiser Permanente Northwest Center for Health Research. Thank you for taking the time today to help us with the IMPULSS study. Is this still a good time to talk? [If not, re-schedule]***

***As you might recall from the invitation letter, [Insert Institution Name] is working with other health care systems across the country to study how patients learn about an inherited colorectal cancer condition known as Lynch syndrome. You were invited to be interviewed because you [recently had a colonoscopy/were recently diagnosed with [type] cancer]. Even though you may not have Lynch syndrome and even though you may never have been screened for Lynch syndrome, your feedback will help us improve how we talk to patients about Lynch syndrome and getting screened. We expect the interview to take 45-60 minutes.***

***I will be recording the interview to make sure I don’t miss anything. Everything you tell me today will be kept confidential. In future publications we may use any quotes that explain a point particularly well, but nothing we use will identify you personally in any way. You do not have to answer any questions you don’t want to, and you can stop at any time.***

***Do you have any questions before we get started? [answer questions] May I get your verbal permission to record? [record answer]***

**Opening question and general awareness of hereditary cancer and Lynch syndrome**

1. Please share with us what the doctor has told you about your recent colonoscopy procedure?
   1. Probe (if it applies): What were you told about the outcome of that procedure
   2. Probe (if it applies): What was the type of cancer?
   3. Probe (if it applies): What stage of cancer do you have?
2. What (if anything) do you already know about genes that can increase a person’s risk for cancer?

***Narrative: “Most of the time cancers are not caused by inheriting a gene that places a person at a higher risk for cancer; however, some cancers are.” [if needed: explain about 5-10% are]***

1. Have you ever been screened for or told you have an inherited gene that can increase cancer risk?
   1. Probe (If needed): For example, has your blood or saliva ever been tested for inherited genes that can increase cancer risk? If yes, did that test identify that you have an inherited gene that can increase cancer risk?
   2. **If YES**, ask participant to describe how this came about and what they know about the identified inherited gene
2. Have you ever heard of Lynch syndrome before this study? [**If No, skip to LS description narrative below**]
3. [**If YES**] Please share with us what you know about it?

**Description of Lynch syndrome in general**

***Narrative: “Lynch syndrome is caused by inheriting a gene that does not work properly. A person with Lynch syndrome has a much higher risk for colorectal cancer than the average person. If a person has the gene for Lynch syndrome, they are at higher risk for additional and/or new cancers, and there are steps they can take to help prevent these cancers.”***

1. What is your reaction to the information I just shared?
2. Probe: What are some reasons you might want to find out if you have Lynch syndrome?
3. Probe: What might be some reasons why you might not [or do not] want to know if you have Lynch syndrome?

***Narrative: “Knowing a person has the inherited gene for Lynch syndrome can tell us more about a current cancer and available treatment options.” How does this information affect…***

1. Probe: What are some reasons you might want to find out if you have Lynch syndrome?
2. Probe: What are some reasons why you might not [or do not] want to know if you have Lynch syndrome?

***Narrative: “Many professional organizations (like the CDC) recommend that all patients newly diagnosed with colorectal cancer have a lab test run on their tumor or biopsy to see if they might have Lynch syndrome and should undergo additional testing. However, some hospitals may not screen all patients for Lynch syndrome routinely, if at all; and we really don’t know enough about what patients think about Lynch syndrome screening. This is why your opinions are so important.*”**

1. What are your thoughts about having these lab tests done on your tumor/biopsy to screen for Lynch syndrome?
2. What are some reasons you think it would be important or not important to have the information that your tumor might be related to Lynch Syndrome
3. If you did want this type of information from tumor screening, how would you expect to get the results back?
4. Probe on preferences: phone call, letter, secure email, in-person visit, other, etc.
5. Probe on if it matters or not who shares the result: PCP, genetic specialist, oncologist, other
6. What kinds of concerns might you have about your tumor being screened for Lynch syndrome?
7. What kinds of concerns, if any, might you have about the documentation of tumor screening results in your medical record?
8. Probe: Are you more concerned about tumor test results being in your medical record than other types of medical information? Please explain.

***Narrative: “If the lab tests on your tumor/biopsy show you MIGHT have Lynch Syndrome, this is just the first step in the process. This is because testing a tumor does not tell us for sure if a person has Lynch syndrome, it can just tell us who is more likely to have Lynch syndrome. A SECOND test of the genes for Lynch syndrome is needed to see if the person does or does not have Lynch syndrome. Most places give patients the choice of whether or not they want to have this SECOND test to find out for sure if they have Lynch syndrome. This SECOND lab test typically requires a separate appointment to discuss Lynch syndrome and to take the blood sample for genetic testing.”***

1. What do you think about this process of having to get a second test to confirm if you have Lynch syndrome?
2. Probe: Would you get this test to confirm? Why or why not?
3. What information would be important for you to have in making your decision to pursue the additional genetic test to determine if you have Lynch syndrome?
4. Probes: cost, how ‘certain’ a result is, timing of when testing/result would occur or be known, etc.
5. [*Note: If cost comes up, ask these probes if not already asked]:* What impact would the cost of this test have on your decision? Do you usually have concerns about your insurance costs for different tests? Please describe.

***Narrative: “Many people choose to have the SECOND (genetic) test to learn if they have Lynch syndrome, but some people may choose to not have that second test.”***

1. Given what we just described, why might you want to have the SECOND (genetic) test to confirm if you have Lynch syndrome?
2. Probe on if this differs from what they stated in above section or if other reasons come up / do they perceive value in knowing the information from the blood test
3. Probe on whether the timing of the test (right after a diagnosis of colorectal cancer) would impact their decision to undergo additional testing
4. Probe on how and from whom they’d like to receive results on the additional genetic test
   - 1. Probe on preferences: phone call, letter, secure email, in-person visit, other, etc.
     2. Probe on if it matters or not who shares the result: PCP, genetic specialist, oncologist, other
5. What might keep you from getting the SECOND test to confirm if you have Lynch syndrome? Please explain.

a. Probe if say Yes/Give reasons: Could anything change your mind about not wanting to get the blood test to confirm if you have Lynch syndrome? (e.g. more information, speaking with specialist or provider, recommendation from provider, family/friend encouragement, insurance/benefits, help with costs, cancer fear, etc.)

1. If you didn’t have to have a separate appointment for the SECOND test, how would that affect your decision to have the test or not? Please explain.
   1. Probe: Would you be more likely to get the test done? Please explain.
   2. Probe: What other things might impact whether you have the follow-up blood test or **not?**

a. Probe: Please describe why did you choose that number.

**Overall entire process – tumor screening and learning about Lynch syndrome**

***Narrative: “After what we’ve just been talking about regarding Lynch syndrome screening…***

If your health system, [insert name] were to regularly screen colorectal cancer patients for Lynch syndrome, how would you feel about it?

a. Probe: Would you want your health system [insert name] to do that? Why or why not?

1. What do you want your health system or hospital to know if they were thinking about or are already doing Lynch syndrome tumor screening?
2. Is there anything else you would like to share about screening for Lynch syndrome?

***Narrative:* *“Now I want to talk a little about what happens after a person is diagnosed with Lynch syndrome. When a diagnosis of Lynch syndrome is confirmed by genetic testing, there are several actions that are recommended. These may include having a colonoscopy every 1 to 2 years for the rest of a person’s life (instead of every 5-10 years for the average person). A colonoscopy can help to find polyps and remove them before they become cancerous or it can find the cancer early when it is easier to treat.”***

1. If testing found you had Lynch syndrome, How would/do you feel about the recommendation to receive colonoscopy exams more often? [*Note: Make modification to this question if it is learned that the patient does have LS (somehow got through screening)]*
2. What would help you to follow the colonoscopy recommendations?

a. Probe: reminders/tracking from the system/ more information from health care system/providers, other

1. What might prevent you from following this recommendation? Please describe.
2. Does knowing this recommendation change how you feel about the importance of tumor screening for Lynch syndrome? If yes, why? If no, why not?

***Narrative: “When a person is diagnosed with Lynch syndrome, it means that other people in the family may also have Lynch syndrome. First degree relatives, like brothers, sisters and children, have a 50% chance of having inherited the gene that causes Lynch syndrome. Because they can do things to prevent cancers, healthcare providers recommend that people with Lynch syndrome share this information so that other family members can choose if they want to have genetic testing.”***

1. Do you think your family members would want this information? Why or why not?
2. What might you want to tell family members about Lynch syndrome?

3) Which family members would you tell? Why is that? [*possible prompts*: important to share for their own health planning; my responsibility to share; possibly prevent colon or other cancers; important for generational family knowledge/history, other, etc.]

4) Are there family members you wouldn’t tell?

a. Probe: What might keep you from telling family members?

[*possible prompts:* might generate fear/worry; do not have close relationship or contact with member; not the “right time” in their life to tell them; cost barriers; health insurance barriers, other, etc.]

5) If you were [or have told] to tell your family member(s) about a possible risk of Lynch syndrome, how would you go about sharing this information? [e.g. in-person, by phone, by email, share/mail information, etc.]?

a. Probe: Would you share information in different ways with different family members? Please describe.

6) What other things might help you talk to family members about possible risk of Lynch syndrome? [e.g. letters, pamphlets, videos, things you can post, laboratory information]

a. Probe: Would you want your healthcare provider/system [name institution] to help talk with your relatives about Lynch Syndrome?

b. Probe: If so, how would you like their help?

**CLOSING**

1) Given what you heard so far, what are your general concerns (if any) about screening for Lynch syndrome? [**note**: after response, probe on following if not brought up]

a. Any general concerns about learning or knowing this information?

b. Any additional concerns about privacy/ documentation of tumor results or LS in medical record?

c. Any additional concerns about the follow up genetic test to confirm Lynch syndrome?

d. Any additional concerns about engaging in more frequent colonoscopy surveillance?

e. Any additional concerns about the cost of the tumor screening for Lynch syndrome, the cost of the genetic testing, or the cost of the follow-up colonoscopy recommendation?

1. Before we ask you some background questions, any final comments you would like us to know about this topic of screening for Lynch syndrome – either that we did not discuss or that you would like to emphasize again as important for us to know or think about?

**DEMOGRAPHICS**

***We have just a few more questions for you about your background. These questions help us understand how medical systems can better communicate about screening for Lynch syndrome.***

What type(s) of health insurance coverage do you have? (**answer all that apply**)

- Private insurance (for example, [INSTITUTION] Health Plan or Blue Cross Blue Shield)
- Medicaid
- Medicare
- Tricare/military
- Don’t know
- None
- Other (specify)_______________________________
- Prefer not to answer (DO NOT READ)

[Note: If participant does not know health insurance carrier, obtain data from the EHR]

Because we are talking about Lynch syndrome which has implications for your family members, it would help us to understand how many of your family members also receive healthcare from [INSTITUTION]. Which of your family members related to you by blood also receive their healthcare from [INSTITUTION]? (answer all that apply)

- Child(ren)
- Parent(s)
- Grandparent(s)
- Sibling(s) (brother/sister)
- None
- Other (specify) ______________________________________
- Prefer not to answer (DO NOT READ)

[Note: If participant mentioned a prior cancer diagnosis during their interview, ask:]

You talked about a cancer diagnosis you had previously. What was the diagnosis?

Can you tell me when that was? (Record in month/year if available)

Please tell me which of the next categories you would use to describe your background? You may answer more than one if that applies for you. (STATE ALL CATEGORIES)

⬜0 American Indian, Native American, or Alaska Native

⬜1 Asian

⬜2 Black or African American

⬜3 Native Hawaiian/Pacific Islander

⬜4 White or European American

⬜5 Middle Eastern or North African/Mediterranean

⬜6 Hispanic/Latino(a)

⬜7 None of these fully describe me (Please describe): ____________________

⬜8 Don’t know

⬜9 Prefer not to answer (DO NOT READ)

Are you currently married or living with a partner?

- Yes
- No
- Prefer not to answer (DO NOT READ)

Are you currently working for pay?

- Yes
- No
- Retired
- Prefer not to answer (DO NOT READ)

Please select the range closest to your total combined yearly **household** income before taxes. (Select one)

- < $15,000
- $15,000 - $30,000
- $30,001 - $50,000
- $50,001 - $75,000
- $75,001 - $100,000
- $100,001 - $150,000
- $150,001 - $200,000
- > $200,000.
- Don't know
- Prefer not to answer (DO NOT READ)

Including yourself, how many people are supported by that income? ________

- Prefer not to answer (DO NOT READ)

What is the highest level of education that you have completed? (Select one)

- Grade school/junior high
- Some high school
- High school graduate
- Trade/technical/vocational school
- Some college
- College graduate
- Post graduate work or graduate degree
- Don’t know
- Prefer not to answer (DO NOT READ)

How confident are you filling out medical forms by yourself? [Note: not including times when you need help filling out forms due to vision problems.] (Select one)

- Extremely
- Quite a bit
- Somewhat
- A little bit
- Not at all
- Prefer not to answer (DO NOT READ)

When people tell you the chance of something happening, do you prefer they use words or numbers? For example, a word example might be “There is a slight risk of something happening” or a number example might be “There is a 5% chance of something happening”.

- Prefer words
- Prefer numbers
- No preference / both are fine
- Prefer not to answer (DO NOT READ)

Thank you for taking the time to talk with me today. We’d like to send you a $25 Amazon gift code to thank you for your time. Would you like me to send this to your email or to a home address?

- Email address: (Fill in email address)
- Home (confirm mailing address)

**THANK YOU!**

**Additional File 1:** IRB approved interview guide (word doc, 39 KB). Interview questions asked of participants.
