# Supplementary material for: “Go ahead and screen” - advice to healthcare systems for routine lynch syndrome screening from interviews with newly diagnosed colorectal cancer patients
Source: Hered Cancer Clin Pract. 2023 Nov 17;21:24. doi: 10.1186/s13053-023-00270-4 (PMC10657118; doi:10.1186/s13053-023-00270-4)
Supplement: Supplementary file 2 — Additional file 2. Topical areas and related codes (Supplementary material for publication). [file 13053_2023_270_MOESM2_ESM.docx]

| **Supplementary Table: Topical Areas for Content Summary Process and the Related Codes*** |
| --- |
| **Topical area: *Patient history / background*** |
| **Colonoscopy/Cancer history:** Comments about colonoscopy outcome, reaction to cancer diagnosis, cancer stage and related surgery/treatment, personal medical history, and family medical history.  **Stories:** Code any patient story (narrative description of any length) about experiences with diagnosis or treatment of CRC**, prior conditions, someone in their family with a genetic condition or health experience, etc. Or anything coder deems “story-like” that is relevant to participants beliefs or orientation on the topic. |
| **Topical area: *Hereditary knowledge / understanding*** |
| **General hereditary baseline knowledge:** Any comments made regarding prior knowledge about genes that can increase a person’s risk of cancer; does not have to be LS*** based knowledge but general knowledge including “nothing” or “I don’t know”. May at times be double coded with LS baseline knowledge/experience code.  **LS baseline knowledge/experience:** Comments on whether participant has any prior knowledge/awareness of LS or inherited genes for CRC; and what that awareness/knowledge is (including “no prior knowledge”). Also includes any description of whether participants’ have ever been screened for any inherited conditions, and how this came about – based on participants’ understanding/account. |
| **Topical area: *General reasons to learn about LS status*** |
| **Reaction/reasons regarding general LS information:** Participants initial reaction to the interview guide narrative describing LS, including reasons they might want to know or learn if they have LS. Including “I don’t know” responses.  **Changes to reasons re LS:** Comments from participants about whether their reasons for knowing about LS diagnosis is altered or not when they understand a LS identification can inform cancer prognosis, treatment plans, and follow-up care including more frequent colonoscopies or removal of ovaries, etc. Code even if they say it does not alter their reasons/reactions. |
| **Topical area: *General reasons to NOT learn about LS status*** |
| **Reasons to not know about LS**: Reaction and reasons as to why participants don’t want to or may not want to learn if they have LS after hearing narrative description. Including comments about being unsure regarding any reasons.  **Changes to reasons re LS:** Comments from participants about whether their reasons for knowing about LS diagnosis is altered or not when they understand a LS identification can inform cancer prognosis, treatment plans, and follow-up care including more frequent colonoscopies or removal of ovaries, etc. Code even if they say it does not alter their reasons/reactions. |
| **Topical area: *Reasons to learn / obtain tumor screening (step 1)*** |
| **Reaction/reasons regarding tumor screening:** Participants initial reaction to narrative explaining tumor screening (first step) for LS and reasons they feel it is important to learn if their tumor is related to LS. Including “I don’t know” responses.  **Tumor screening concerns:** Any concerns brought up by participants regarding obtaining, learning about, or documenting tumor screening results. Includes response to probe re: concern or not of having tumor screening result in medical record at all or as compared to other conditions. May be double coded with “privacy/documentation” code. |
| **Topical area: *Reasons to NOT learn / obtain tumor screening (step 1)*** |
| **Reason to not know regarding tumor screening**: Reaction and reasons as to why participants don’t want to know about or may not want to learn if their tumor is related to LS. May be double coded with other barrier codes or the tumor screen concern code (e.g. cost, certainty, etc.). Include comments about being unsure regarding reasons.  **Tumor screening concerns:** Any concerns brought up by participants regarding obtaining, learning about, or documenting tumor screening results. Includes response to probe re: concern or not of having tumor screening result in medical record at all or as compared to other conditions. May be double coded with “privacy/documentation” code. |
| **Topical area: *Reasons to learn / obtain genetic test (step 2)*** |
| **Reaction/reasons regarding genetic test:** Participants initial reaction to narrative explaining genetic blood test (second step) needed to confirm LS Dx, and reasons they feel it is important learn this information. Including “I don’t know” responses.  **Decision-making factors regarding blood test:** Participants comments on information they would want to know or understand to help them decide whether to obtain additional blood test, such as cost, certainty, hand-outs explaining the process, timing, consent, insurance, having to do an additional appointment, or “nothing”. May be double coded with specific codes on things like cost or consent, or preferred mode/timing etc.  **Changes to blood test reasons:** Any comments from participants re factors that could change whether they would or would not obtain the additional genetic blood test. Code even if they say it does not alter their reasons/reactions. |
| **Topical area: *Reasons to NOT learn / obtain genetic test (step 2)*** |
| **Reasons to not know re genetic test:** Reaction and reasons as to why participants don’t want to know or may not want to obtain the genetic blood test (second step) or learn this information. May be double codes with other barrier codes or decision-making factor code. Include comments about being unsure re any reasons.  **Decision-making factors regarding blood test:** Participants comments on information they would want to know or understand to help them decide whether to obtain additional blood test, such as cost, certainty, hand-outs explaining the process, timing, consent, insurance, having to do an additional appointment, or “nothing”. May be double coded with specific codes on things like cost or consent, or preferred mode/timing etc.  **Concerns/barriers regarding additional blood test:** General comments about concerns, fears, barriers, or additional questions participants may have re obtaining or learning results of additional genetic blood test.  **Changes to blood test reasons:** Any comments from participants re factors that could change whether they would or would not obtain the additional genetic blood test. Code even if they say it does not alter their reasons/reactions. |
| **Topical area: *General concerns and questions regarding LS screening*** |
| **Tumor screening concerns:** Any concerns brought up by participants regarding obtaining, learning about, or documenting tumor screening results. Includes response to probe re: concern or not of having tumor screening result in medical record at all or as compared to other conditions. May be double coded with “privacy/documentation” code.  **Concerns/barriers re additional blood test:** General comments about concerns, fears, barriers, or additional questions participants may have re: obtaining or learning results of additional genetic blood test. May be double coded with other specific codes like “cost” or “consent” or “certainty.”  **Cost and insurance concerns/questions:** Comments, questions, or concerns about cost of tumor screening, genetic blood test, or follow up surveillance – includes comments from participants in asking interviewers about cost/ insurance coverage. May include concerns about insurance coverage loss or rates going up because of LS diagnosis.  **Privacy and documentation concerns/questions**: Comments, questions, or concerns about the results of tumor screening and/or blood test results in medical record – any comments about concern or lack of concern for privacy and documentation. May include comments about insurance discrimination fears.  **Consent concerns/questions:** Comments, questions, or concerns about if and when consent should be obtained or if consent is not necessary.  **Accuracy of testing concerns/questions:** Comments, questions, or concerns about how accurate the screening is for LS or questions on if the diagnosis/result for LS can change over time as science or knowledge evolves. Any concerns expressed about how “certain” the result is or not.  **Final concerns**: Any final concerns shared at end of the interview about screening for LS, including “none”. May be double coded with specific concerns/questions on other issues (e.g. cost). |
| **Topical Area: *Communication and timing of LS results*** |
| **Preferred mode/timing of obtaining tumor screening results:** Participants description of how they would hypothetically like to receive tumor screening results (in-person, letter, call, email, etc.), when they would like to learn this information, and from whom (PCP****/Specialist) or if this matters to or not to the participant. May be double coded at times with decision-making factors.  **Preferred mode/timing of obtaining genetic test results:** Participants descriptions of how they would hypothetically like to receive genetic test results (in-person, letter, call, email, etc.), when they would like to learn this information (e.g. right after diagnosis/surgery or later), and from whom (PCP/Specialist) or if this matters or not to the participant. May be double coded at times with decision-making factors.  **Advice to health system:** What participants would want their health system to know or do in terms of supporting LS screening. May be double coded with other related codes. |
| **Topical Area: *Role of Health System*** |
| **Health system screen reaction:** Descriptions of how participants would feel about their health system screening for LS and if they feel their health system should do this (or not) and why. May be double coded with other related codes.  **Advice to health system:** What participants would want their health system to know or do in terms of supporting LS screening. May be double coded with other related codes.  **Support in sharing with Family:** Participants comments on whether they would want assistance from their health plan when informing family members of LS status, and in what form – what additional support or information would the participant find helpful (e.g. hand-outs, call from genetic specialists, etc.). |
| **Topical Area: *Colonoscopy surveillance reaction*** |
| **Colonoscopy surveillance reactions:** Comments and reactions to more frequent colonoscopy cancer screening recommendations based on possible LS diagnosis, and why they might follow through on this – what would help, such as reminders.  **Colonoscopy surveillance barriers:** What participants believe would actually or hypothetically be barriers to engaging in more frequent colonoscopies or other surveillance recommendations following a LS diagnosis.  **Changes to reasons due to surveillance recommendations:** Comments from participants on whether their reasons for wanting to learn/know about a LS diagnosis or being tested for it is altered or not when they understand surveillance recommendations post LS diagnosis. Code even if indicate ‘no change’. |
| **Topical Area: *Future plans and final thoughts*** |
| **Future plans:** Any actions participants say they plan to take but haven’t yet (e.g. obtaining additional testing, discussing with family, follow up with providers, etc.) regarding LS screening and/or diagnosis. Can include actual plans or also something more hypothetical, e.g. *if I was in X situation I think I would do Y.*  **Final thoughts:** Any additional comments at end of interview about importance, need for, or reaction to screening for LS or any other comment made at the end of interview deemed as important or final to participant. |

**Codes were iteratively reviewed, and may have been reviewed for more than one topical area as part of the overall content analysis and integration process; ** CRC=Colorectal cancer; *** LS=Lynch syndrome; **** PCP=Primary care provider*
